# A Birth Cohort Follow-Up Study on Congenital Zika Virus Infection in Vietnam

**DOI:** 10.3390/v15091928

**Published:** 2023-09-15

**Authors:** Michiko Toizumi, Cuong Nguyen Vu, Hai Thi Huynh, Masafumi Uematsu, Vy Thao Tran, Hien Minh Vo, Hien Anh Thi Nguyen, Mya Myat Ngwe Tun, Minh Xuan Bui, Duc Anh Dang, Hiroyuki Moriuchi, Lay-Myint Yoshida

**Affiliations:** 1 Department of Pediatric Infectious Diseases, Institute of Tropical Medicine, Nagasaki University, Nagasaki 852-8523, Japan; lmyoshi@nagasaki-u.ac.jp; 2 Khanh Hoa Health Service, Nha Trang 650000, Vietnam; cuongnv2005@gmail.com (C.N.V.); huynhthihai46@gmail.com (H.T.H.); bsminhnt@gmail.com (M.X.B.); 3 Department of Ophthalmology, Nagasaki University Hospital, Nagasaki 852-8501, Japan; uematsu@nagasaki-u.ac.jp; 4 Khanh Hoa General Hospital, Nha Trang 650000, Vietnam; tranthaovy1981@gmail.com (V.T.T.); doctorhien80@yahoo.com (H.M.V.); 5 National Institute of Hygiene and Epidemiology, Hanoi 100000, Vietnam; hienanh75@yahoo.com (H.A.T.N.); dangducanh.nihe@gmail.com (D.A.D.); 6 Department of Virology, Institute of Tropical Medicine, Nagasaki University, Nagasaki 852-8523, Japan; myamyat@tm.nagasaki-u.ac.jp; 7 Center for Vaccines and Therapeutic Antibodies for Emerging Infectious Diseases, Shimane University, Izumo 693-0021, Japan; 8 Department of Pediatrics, Graduate School of Biomedical Sciences, Nagasaki University, Nagasaki 852-8501, Japan; hiromori@nagasaki-u.ac.jp

**Keywords:** Zika virus, congenital Zika virus infection, congenital Zika syndrome, follow-up, child development, birth cohort, Vietnam, Asia

## Abstract

We assessed the development, sensory status, and brain structure of children with congenital Zika virus (ZIKV) infection (CZI) at two years and preschool age. CZI was defined as either ZIKV RNA detection or positive ZIKV IgM and neutralization test in the cord or neonatal blood. Twelve children with CZI born in 2017–2018 in Vietnam, including one with Down syndrome, were assessed at 23–25.5 months of age, using Ages and Stages Questionnaire (ASQ-3), ASQ:Social-Emotional (ASQ:SE-2), Modified Checklist for Autism in Toddlers, automated auditory brainstem response (AABR), and Spot Vision Screener (SVS). They underwent brain CT and MRI. They had detailed ophthalmological examinations, ASQ-3, and ASQ:SE-2 at 51–62 months of age. None had birthweight or head circumference z-score < −3 except for the one with Down syndrome. All tests passed AABR (*n* = 10). No ophthalmological problems were detected by SVS (*n* = 10) and detailed examinations (*n* = 6), except for a girl’s astigmatism. Communication and problem-solving domains in a boy at 24 months, gross-motor area in a boy, and gross-motor and fine-motor areas in another boy at 59–61 months were in the referral zone. Brain CT (*n* = 8) and MRI (*n* = 6) revealed no abnormalities in the cerebrum, cerebellum, or brainstem other than cerebellar hypoplasia with Down syndrome. The CZI children were almost age-appropriately developed with no brain or eye abnormalities. Careful and longer follow-up is necessary for children with CZI.

## 1. Introduction

Zika virus (ZIKV) is an arthropod-borne virus in the family Flaviviridae, genus *Flavivirus*, transmitted primarily by *Aedes* mosquitoes. A potential link between maternal ZIKV infection and congenital syndrome was suggested in a large ZIKV epidemic in the Americas in 2015 and was later confirmed. Most ZIKV infections in pregnancy are asymptomatic, but maternal-fetal transmission can occur in 7–30% of maternal infections [1,2]. Children exposed to ZIKV in utero may develop a spectrum of structural anomalies and functional disabilities secondary to central nervous system damage, known as congenital Zika syndrome (CZS), with the most common clinical feature being microcephaly. Normocephalic children born to mothers with ZIKV infection during pregnancy, and with no observable Zika-associated birth defects, may also present later with neurodevelopmental delay or postnatal microcephaly [3]. Recently, studies in the Americas have reported delayed neuro-motor development of children with congenital Zika infection (CZI) or congenital Zika syndrome (CZS) in the two-to-three-year follow-ups [4,5,6,7,8,9,10,11].

Two major lineages of ZIKV have been identified: African and Asian lineages. The Asian lineage is responsible for severe diseases in the Americas, whereas the African lineage has not been associated with severe diseases or epidemics. An animal model study reported that the African lineage has higher neurovirulence than the Asian lineage and that virulence is determined by viral structural genes [12]. Comparative studies of two strains from the Asian lineage, an emerging American strain and a less-pathogenic Asian strain, and an African strain showed weaker and delayed innate immune responses to the American strain, which might contribute to the differential pathogenesis of the two strains of the Asian lineage and the African lineage [13,14].

Following the epidemic in the Americas, active surveillance was conducted for ZIKV infections or circulation in Southeast Asia, where *Aedes* mosquitoes are endemic. The evidence has suggested that ZIKV has been circulating in Southeast Asia for more than a decade and yet cases of congenital ZIKV infection remain sparsely documented [15]. Therefore, there are very few reports from Asia on post-infancy neurodevelopment and sensory deficits in children with CZI [16], especially in those who are apparently normal at birth or in infancy.

In this study, we aimed to follow up on children born with CZI and assess their neurodevelopmental, ophthalmological, and otological status at two years and preschool age in Vietnam.

## 2. Materials and Methods

### 2.1. A Birth Cohort and Neonates Suspected of Congenital Infection

We launched a birth cohort study and conducted a survey of neonates suspected of having congenital infections simultaneously at Khanh Hoa General Hospital (KHGH) in Nha Trang, the capital city in Khanh Hoa province, south-central Vietnam [17,18]. KHGH is a provincial hospital with approximately 6000 deliveries per year. For the birth cohort study, we enrolled babies born at KHGH from women aged ≥ 18 years living in 16 target communities in Nha Trang between July 2017 and September 2018. Babies born to women with multiple births, stillbirths, or serious complications before pregnancy were excluded. For the survey of babies born suspected of congenital infection, we enrolled neonates born at KHGH from women who had any two of the following during the pregnancy: fever, rash, arthralgia/arthritis, lymphadenopathy, and conjunctivitis, and neonates born at or referred to KHGH who had any of the following: severe small-for-dates (<the 1st percentile of birthweight for each gestational age estimated from the 2009–2010 birth cohort in the same study area [19,20]), suspected meningoencephalitis (bulging or enlarged anterior fontanelle, lethargy, or convulsions), microcephaly (head circumference < 30 cm), hydrocephalus or ventriculomegaly, congenital heart disease, suspected hearing loss (lack of Moro reflex in response to auditory stimuli or failure to automated auditory brainstem response [AABR] test if it is completed), cataract, glaucoma, hepatosplenomegaly, hepatitis, jaundice (onset within 24 h after birth or requiring exchange transfusion), purpura and/or petechiae, lymphadenopathy, anemia (Hb < 13 g/dL), and thrombocytopenia (<150,000/µL). Neonates with confirmed chromosomal abnormalities or well-known congenital anomalies related to genetic defects were excluded. We collected demographic and socioeconomic information from mothers and perinatal and neonatal information, including the presence of the symptoms listed above, from medical charts using a standardized data collection form. The process was conducted by two trained research staff nurses at KHGH and two trained field workers from Khanh Hoa Health Service, under the supervision of a research clinician in KHGH.

Cord or peripheral blood samples were collected immediately after delivery or upon admission from the enrolled babies. Saliva was collected between feedings. Plasma samples were tested for ZIKV, dengue virus, and Japanese encephalitis virus-specific immunoglobulin M (IgM), and flavivirus-specific immunoglobulin G (IgG) was tested using an in-house enzyme-linked immunosorbent assay. The plasma samples were also tested by a 50% focus reduction neutralization test for ZIKV, four serotypes of dengue virus, and Japanese encephalitis virus. RNA specimens extracted from the plasma samples were tested for ZIKV using quantitative reverse transcription polymerase chain reaction (qRT-PCR) to detect CZI [18]. We also tested the plasma samples for rubella-specific IgM, IgG, and cytomegalovirus-IgM. The saliva and whole blood samples were tested for rubella virus RNA and cytomegalovirus DNA using qRT-PCR and real-time PCR, respectively [14]. We regarded a case as CZI in either case of ZIKV RNA detection in the cord or peripheral blood plasma or positive ZIKV IgM and neutralization test. The latter was considered significant when it was positive only against ZIKV, or when the neutralizing antibody titer against ZIKV was ≥4 times higher than those against other flaviviruses [18].

We enrolled 2013 babies in the birth cohort study and 150 babies in the survey on suspected congenital infections. Among them, CZI was detected in 11 (0.5%) from the birth cohort but not in the suspected congenital infection cases [18]. None of them had congenital rubella or cytomegalovirus infection [17]. No mothers were tested for ZIKV during pregnancy. One boy enrolled in the survey for suspected congenital infection was later excluded because he had Down syndrome. However, he was diagnosed with CZI and was included in this follow-up study on children with CZI with his mother’s consent because he also could have CZI-associated symptoms such as posterior eye problems. A total of 12 children with CZI were followed up for further clinical examinations.

### 2.2. Twenty-Four Month-Follow-Up with Eye, Hearing, and Developmental Screening

We visited each of the participants at home when they were between 23 and 25 months and 15 days old, following the target age of the 24-month questionnaire of the Ages and Stages Questionnaire^®^, third edition (ASQ-3). The children’s body weight, height, and head circumference were measured. Developmental milestones, medical history, and family information were collected from their mothers using a questionnaire. Developmental assessment, hearing screening, and eye screening were conducted during the same home visits as below.

Developmental, social, and emotional difficulties were screened using the 24-month questionnaire of ASQ-3, the 24-month questionnaire of Ages and Stages Questionnaires^®^: Social-Emotional, Second Edition (ASQ:SE-2), and the Modified Checklist for Autism in Toddlers, Revised, with Follow-Up™ (M-CHAT-R/F) [21]. Vietnamese versions of ASQ-3 and M-CHAT R/F are officially available. Prepublication-Vietnamese ASQ:SE-2 was provided by the manufacturer. The ASQ-3 is a parent-administered, structured questionnaire that includes questions in five domains of child development: communication, gross motor skills, fine motor skills, problem-solving skills, and personal–social skills. Sum scores in each of the five developmental areas were categorized into three: typical development (pass) with a score < 1 standard deviation below the mean, monitoring zone with a score ≥ 1 and <2 standard deviations below the mean, and referral zone with a score ≥ 2 standard deviations below the mean [22]. ASQ:SE-2 is a parent-completed questionnaire focused solely on social and emotional development in young children. Score of ASQ:SE-2 also has a designated cutoff, which tends to be closer to the 80th percentile point. Children with scores above the cutoff are in the referral zone and those from the 65th percentile point up to the cutoff are in the monitoring zone [23]. Children in the monitoring zone need to be monitored with rescreening in a couple of months and those in the referral zone need facilitated referral for further evaluation and/or services [22,23]. The M-CHAT-R/F is designed to screen for autism spectrum disorders (ASD) in toddlers by asking caregivers to report 23 behaviors related to sensory abnormalities, motor abnormalities, social interchange, early joint attention/theory of mind, early language, and communication.

We used an AABR (Echo-Screen MAAS, Nippon-Koden, Japan) to screen for hearing impairments. Stimuli were presented at 35 and 45 decibels, corresponding to normal hearing levels.

Spot Vision Screener (SVS) (Welch Allyn, Skaneateles Falls, NY, USA) was used to screen pupillary diameter, ocular alignment, and estimated binocular refraction. The SVS provides an interpretation of either “all measurements within range” or “complete eye exam recommended”. When the device is unable to evaluate a subject, it will post “pupils too small”, “pupils not found”, or “out of range” [24].

### 2.3. Neuroimaging, Detailed Eye Examinations, and Developmental Assessments

We invited the participants to KHGH, and the children underwent brain imaging using computed tomography (CT) in 2020 and magnetic resonance imaging (MRI) in 2022. Ophthalmological examinations were conducted by ophthalmologists when children visited KHGH to undergo MRIs. A pediatric neurologist interpreted the images. The ophthalmologists examined the children’s visual acuity, ocular alignment, refraction, intraocular pressure, and the anterior and posterior segments of the eye. Bilateral retinal imaging was performed after pupil dilation using a handheld fundus camera (Optmed Aurora; Optomed, Oulu, Finland). Sedative drugs were used during brain imaging and fundoscopy by a pediatrician following the KHGH guidelines. Before ophthalmological examinations, we measured the child’s body weight, height, and head circumference and screened for developmental difficulties using the 54-month or 60-month questionnaire of ASQ-3 and the 48-month or 60-month questionnaire of ASQ:SE-2 depending on their age. 

Z-scores of children’s weight, length (height), weight for length (height), and head circumference at birth and the follow-ups were calculated using the WHO’s child growth standards [25]. The WHO’s motor development milestones were used to assess the children’s gross motor milestones [26]. 

This study was approved by the Institutional Ethical Review Boards of the National Institute of Hygiene and Epidemiology, Hanoi (IRB-VN01057-30/2015) and the Institute of Tropical Medicine, Nagasaki University (160908158). Written informed consent was obtained from all the guardians before participation.

## 3. Results

Among the 12 children with CZI, we could follow up with 11 at 23–25.5 months of age in 2019–2020, eight for brain CT in 2020, and six for MRI and detailed eye examinations at 51–62 months of age in 2022.

### 3.1. Perinatal Findings of Children with CZI

All 12 babies with CZI, six boys and six girls, were born full-term (≥37 gestational weeks) with an Apgar score of 9–10 in five minutes after birth. One boy with Down syndrome had a low birth weight (2200 g, z-score −2.67), microcephaly (30 cm, z-score −3.51), patent ductus arteriosus, and thrombocytopenia (case L in Table 1). One girl born at 37 weeks and 2 days had a birth weight of 2500 g (z-score −1.73) and a head circumference of 31.5 cm (z-score −2.01) (case K). None of the other 10 babies had birth weight, length, head circumference, or weight-for-length less than −2 of the z-score. The mother of another girl (case F) had oligoamnios during pregnancy. The girl had purpura/petechiae, was hospitalized in the pediatric department for one day for observation and was discharged well. None of the 12 mothers reported fever, rash, arthralgia/arthritis, lymphadenopathy, or conjunctivitis during the pregnancy. The details are presented in Table 1.

### 3.2. Sensory Features of Children with CZI

We screened hearing ability using AABR in 10 children at the age of 23–25.5 months. Ten children passed the screening with a response of 35 dB in both ears (Table 2). 

**Figure 1:**
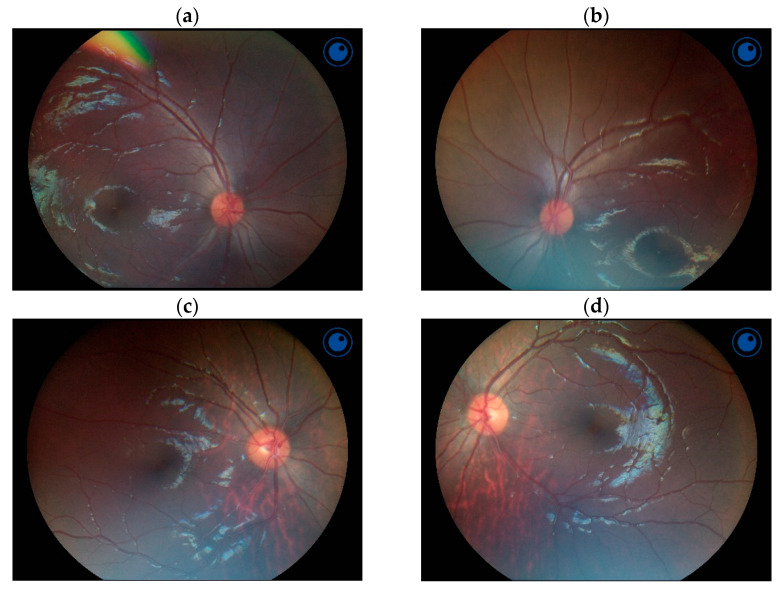
Bilateral fundus photographs of cases A (**a**,**b**) and J (**c**,**d**). Case A was a 62-month-old girl with bilateral astigmatism, and Case J was a 51-month-old boy. None of the children had abnormal findings in the fundus.

We used SVS for ten children when they were 23–25.5 months old: one girl (case A in Table 2) completed the screening with “complete eye exam recommended” because of bilateral astigmatism, five completed it with “all measurements within range,” and four could not complete it with “pupils too small.” In the second ophthalmological examination of six children aged 51–62 months, one girl (case A) had bilateral astigmatism with more than two diopters in the refraction test. Other than that, none of the children showed abnormal findings in the examinations, including fundoscopic findings (Figure 1), such as chorioretinal atrophy and macular pigmentary mottling. All five children tested for visual acuity had a best-corrected visual acuity of 0.7 or higher. We could not perform the second ophthalmological examination in five children; of those, three had SVS measurements all within range and two had not completed SVS measurements at 23–25.5 months of age.

### 3.3. Developmental Milestones and Screening of Children with CZI

Among the 11 children whose development was observed at the age of 23–25.5 months, three children, including one boy with Down syndrome, started sitting without support later than the WHO average plus two standard deviations. However, all of them could walk alone at the age of 13 months or earlier, except for the boy with Down syndrome who could not walk at 24 months of age (case L in Table 3). The twenty-four-month questionnaire of ASQ-3 screened the 11 children and detected one boy (case J) with scores below the referral cut-off (two standard deviations below the mean) in the communication and problem-solving areas and a score in the monitoring zone (≥1 and <2 standard deviations below the mean) in the personal-social area. A girl (case A), a boy (case B), another boy (case G), and another girl (case I) scored in the monitoring zone in the gross motor, personal-social, personal-social, and fine motor areas, respectively. The boy with Down syndrome (case L) had ASQ-3 scores below the referral cut-off in all five developmental areas. Boys J and L, and another boy E, who had scores indicating age-appropriate development by ASQ-3, had scores above the referral cut-off of ASQ:SE-2, which indicates a possible delay in social-emotional development. All 11 children, except for the boy with Down syndrome (case L) had scores 0–1 of M-CHAT-R/F indicating a low risk of autism spectrum disorder. 

We could see six of the 11 children again when they were 51–62 months old. Boy J, who had scores lower than the referral cut-off or in the monitoring zone in several areas of the ASQ-3 and ASQ:SE-2 at 24 months of age, caught up close to the average scores overall and only had scores in the monitoring zone in the gross motor area of the ASQ-3 and ASQ:SE-2. Meanwhile, boy G, who had scores above the monitoring zone in most of the areas at the age of 25 months, fell behind the average with scores below the referral cut-off in the gross motor, fine motor, and monitoring zones in the problem-solving areas at the age of 59 months. His mother worried about his speech impediment, even though his score in the communication area was +0.27 SD of the average. Boy E, who had a possible delay in social-emotional development at 25 months of age, obtained an ASQ:SE-2 score close to the average but below the referral cut-off in the gross motor area. Boy C had scores indicating age-appropriate development in all screenings at 25 months of age but had scores in the monitoring zone in the fine motor area of ASQ-3 and ASQ:SE-2. His mother worried about his brain development.

In terms of anthropology, boy G had a small body mass index and weight for height below −2 standard deviations at 59 months of age, but none of the examined children had microcephaly or small weight/height for age during the follow-up.

### 3.4. Brain Imaging of Children with CZI

Eight children with CZI underwent brain CT in August 2020. The boy with Down syndrome had hypoplasia of the cerebellar vermis; however, the other seven had neither calcification, ventricular enlargement, or any other findings in the large structures of the cerebrum, cerebellum, and brain stem. Six children, excluding the boy with Down syndrome, underwent brain MRI in 2022. None of the six children showed structural abnormalities of the cerebrum or cerebellum, abnormalities of gyrus formation, or cerebrovascular abnormalities on MRI.

## 4. Discussion

We identified 12 children with CZI from a birth cohort and a survey on suspected congenital infection in Nha Trang, south-central Vietnam. We described the perinatal findings and followed up with 11 of them by developmental, ophthalmological, and hearing screening at 23–25.5 months of age, and further examined six of them by developmental screening, detailed ophthalmological examinations, and brain imaging at 51–62 months of age.

None of the mothers of the 12 children in this study reported any Zika-like symptoms during pregnancy. Previous studies from the Americas enrolled mothers infected with ZIKV during pregnancy in the Zika epidemic and 20–46% of them were reported to be symptomatic [5,9]. The original birth cohort was not a cohort born to women with ZIKV infection or fever during pregnancy but a general cohort in the community. Therefore, our results revealed the CZI incidence in the general population and confirmed that most maternal infections are asymptomatic and emphasized the difficulty of suspecting ZIKV infection during pregnancy. However, the incidence could be underestimated considering the possibility of false-negative results; ZIKV can be positive only transiently in fetal blood [27]. Oligoamnios was reported as one of the abnormal findings of prenatal ultrasonography in women diagnosed with ZIKV infection during pregnancy in association with an increased risk of abnormal neonatal findings [28]. Oligoamnios detected in one case in this study might have been due to ZIKV infection; however, the baby had only mild purpura at birth and no developmental or sensory problems in the follow-up until 24 months of age. 

Microcephaly (z-score < −2) associated with low birth weight was observed in a boy with Down syndrome (case L) and a girl (case K). As far as we could follow up with children with CZI until 2 or 6 years of age, none of them developed microcephaly at infancy or toddler age. Brain imaging using CT or MRI did not reveal typical findings in CZS, such as delayed myelination, intracranial calcification, ventriculomegaly, cerebellar hypoplasia, or cortical development abnormalities, which are seen in most children with CZS [29], except for cerebellar hypoplasia in the boy with Down syndrome, which is commonly seen in Down syndrome. Studies on mothers infected with ZIKV during pregnancy in the US territories reported that 4–6% of the babies had microcephaly at birth, and postnatal-onset microcephaly occurred in an additional 1% [30]. In the same study, even among the children who did not have microcephaly detected at birth, 2% had at least one brain anomaly identified [30]. 

We could follow up with 11 children with CZI at 23–25.5 or 51–62 months of age and found that all of them, excluding one with Down syndrome, developed almost appropriately for their age in all the tested developmental areas. However, some of the children were in the monitoring zone or referral zone of each developmental area: communication, gross motor, fine motor, problem-solving, personal-social, and social-emotional at 23–25.5 months of age. At the 51–62-month-old follow-up, one boy whose communication, problem-solving, and social-emotional development were in the referral zone at 24 months of age caught up in each of the areas to age-appropriate levels, while another boy newly dropped into the referral zone of gross motor and fine motor domains. This observation might indicate a possible change in developmental status with age, either improving or deteriorating, in cases born with CZI. Or, it was more likely to be just a noise in the data which can be observed even in typically developing children, indicating no clear and consistent pattern of developmental concerns in children with CZI. In Puerto Rico, children born to mothers with confirmed and probable ZIKV infection during pregnancy were followed up and 20–60% of them were detected with developmental delay in each developmental area, with some change over time, peaking at around 24 and 36 months [5]. Among a cohort in French Guiana, 20–60% of the children with confirmed CZI were in a referral zone of global, motor, cognitive, language, and socio-affective domains of the Child Development Assessment Scale at 3 years of life [9].

In this study, no CZS-like findings were detected, such as pigment mottling or chorioretinal atrophy in the macular region, iris coloboma, lens subluxation, cataracts, glaucoma, or microphthalmia, in the eyes of children with CZI [31]. Visual acuity was well developed among those tested at 51–62 months old. In the Puerto Rican study that followed children born to mothers with ZIKV infection, 54% of the children with CZI had posterior eye abnormalities in retinal images [5]. In the French Guinean study, 13% of children had impaired responses to visual stimuli at 3 years of age [9]. Among children who underwent ophthalmologic evaluation in a follow-up study in the US territories, 2% had at least one eye anomaly identified [30]. 

Overall, the children born with CZI in this cohort study showed no CZS-like features in their eyes and brain imaging, with almost age-appropriate development. They seemed to be milder than those born with CZI following the epidemic in the Americas in 2015–2016. This might be because the ZIKV strains differed from those in Latin American outbreaks or host factors, such as immune status induced by circulating ZIKV. 

A nucleotide sequence analysis of ZIKV samples from pregnant women with RT-PCR-confirmed ZIKV infection from Ho Chi Minh City detected in a ZIKV surveillance from 2016 to 2017 [32] and from those who traveled to Vietnam [33] identified a clade within the ZIKV Asian lineage in the circulation/outbreak in Vietnam [16]. Phylogenetic analysis by Grant et al. inferred the geographical origin of the clade containing Vietnamese, French Polynesian, and Latin American strains as Vietnam (March 2009), and the parent node as Thailand (July 2007) [16]. The prM S139N mutation in the ZIKV genome, which may be responsible for more severe disease outcomes identified in French Polynesia and Latin America, was not detected in the samples from the pregnant women in Ho Chi Minh City [16]. The ZIKV surveillance, based on symptomatic cases, detected an epidemic with 236 cases including pregnant women in 12 provinces in Vietnam; more than 200 were from Ho Chi Minh City and the others from other southern or central areas, in 2016–2017 [32]. ZIKV infection in this study was likely to be related to the epidemic, geographically and temporally, and ZIKV strains might be the same or similar to those from Ho Chi Minh City, although we could not determine the sequence in this study as RNA in the samples was not sufficient for that. Of 21 children live-born to the women with confirmed ZIKV infection in Ho Chi Minh City, three had microcephaly at birth and three had a developmental delay in a follow-up survey at an average age of 30.8 months, although there was no statistically significant difference from the development of the control group [16]. Strains in Vietnam might be less virulent with a low possibility of microcephaly, considering the small number of children reported with CZS for more than a few pregnant women infected with ZIKV in 2016–2017 [34]. However, considering the reports from Ho Chi Minh City [16], a case report of microcephaly with CZI from Dak Lak [35], and some children in this study requiring referral to specialists or monitoring of their development, strains circulating or causing the epidemic in Vietnam could cause adverse pregnancy outcomes and CZS in newborns.

This study had some limitations: stillbirths with CZI were not included, the small number of children we could follow up with, genomic data of infected ZIKV was not available, and no detailed assessment by child developmental specialists for the children in the monitoring or referral zones of development. In addition, we used the Vietnamese version of the development screening tools validated by the manufacturer; however, the cutoff scores were the same as the original scores derived from U.S. normative samples. Therefore, the results might be affected by differences in the cultural and social backgrounds of the Vietnamese population. However, this is the first study in Asia to follow up on children born with CZI detected in the birth cohort, regardless of the mother’s symptoms. It is important to maintain ZIKV surveillance with greater attention to pregnant women and newborn babies in Vietnam. Considering the possible change in the developmental status over time and the unknown long-term influence of CZI, careful and longer follow-up combined with ophthalmological and other examinations is necessary for children with CZI, regardless of symptoms at birth.

## Figures and Tables

**Table 1:** Characteristics of children with congenital Zika virus infection at birth.

ID	Month of Birth	Mother’s Fever, Arthritis, Conjunctivitis during Pregnancy	Finding(s) in Pregnancy	Sex	Gestational Age at Birth	Birth Weight (gram)	z Score	Birth Length (cm)	z Score	Birth Head Circumference (cm)	z Score	z Score for Weight-for-Length	Apgar score 1 min	Apgar Score 5 min	Neonatal Finding(s)
A	Jul-2017	No	None	F	38w0d	2800	−0.99	49	−0.08	32.5	−1.16	−1.35	9	10	None
B	Aug-2017	No	None	M	38w2d	3000	−0.73	48	−1.00	34.5	0.03	0.19	9	10	None
C	Aug-2017	No	None	M	39w3d	3400	0.11	49	−0.47	33	−1.15	0.91	9	10	None
D	Sep-2017	No	None	F	40w1d	3100	−0.29	49.5	0.19	33	−0.74	−0.53	9	10	None
E	Sep-2017	No	None	M	40w4d	3100	−0.52	49.7	−0.10	33.5	−0.76	−0.46	9	10	None
F	Oct-2017	No	**Oligoamnios**	F	40w2d	3600	0.78	50	0.46	34.3	0.36	0.78	9	10	**Purpura**
G	Nov-2017	No	**Threatened premature delivery**	M	40w2d	2800	−1.18	48.5	−0.73	32	−1.94	−0.92	9	10	None
H	Nov-2017	No	**Early amniorrhexis**	F	39w3d	3400	0.36	50	0.46	33.5	−0.32	0.16	9	10	None
I	Sep-2017	No	None	F	39w2d	3100	−0.29	49	−0.08	33	−0.74	−0.20	9	10	None
J	Jul-2018	No	None	M	39w6d	4000	1.26	52	1.12	34.5	0.03	0.69	9	10	None
K	Sep-2018	No	None	F	37w2d	2500	−1.73	46	−1.69	31.5	**−2.01**	−0.57	9	10	None
L	Jul-2017	No	NA	M	40w0d	**2200**	**−2.67**	46	−2.05	**30**	**−3.51**	−1.92	9	9	**Patent ductus arteriosus, thrombocytopenia (42,000/µL), and Down syndrome**

**Table 2:** Sensory features of children with congenital Zika virus infection.

		Examinations at 23–25.5 Months of Age					Examinations at 51–62 Months of Age						
ID	Month of Birth	Sex	Age (m)	AABR (35 db Right)	AABR (35 db, Left)	SVS	Age (m)	Intraocular Pressure (Right)	Intraocular Pressure (Left)	Fundus	Visual Acuity (Right)	Visual Acuity (Left)	Ophthalmological Finding(s)
A	Jul-2017	F	24.7	pass	pass	**Astigmatism (bilateral)**	62.8	19.3	19.8	Normal	0.4 (0.9)	0.3 (0.9)	**Astigmatism (bilateral)**
B	Aug-2017	M	23.9	pass	pass	All measurements within range	61.9	19.3	18.2	Normal	NA	NA	Normal
C	Aug-2017	M	25.0	pass	pass	All measurements within range	61.8	19	18	Normal	1.0 (n.c.)	1.0 (n.c.)	Normal
D	Sep-2017	F	24.6	pass	pass	Not complete (pupils too small)	NA	NA	NA	NA	NA	NA	NA
E	Sep-2017	M	25.2	pass	pass	Not complete (pupils too small)	61.3	14	15	Normal	1.0 (n.c.)	1.0 (n.c.)	Normal
F	Oct-2017	F	24.2	pass	pass	All measurements within range	NA	NA	NA	NA	NA	NA	NA
G	Nov-2017	M	25.2	pass	pass	Not complete (pupils too small)	59.5	10.3	13.4	Normal	0.9 (n.c.)	0.7 (n.c.)	Normal
H	Nov-2017	F	25.0	pass	pass	All measurements within range	NA	NA	NA	NA	NA	NA	NA
I	Sep-2017	F	25.4	NA	NA	All measurements within range	NA	NA	NA	NA	NA	NA	NA
J	Jul-2018	M	24.8	pass	pass	NA	51	18.1	17.4	Normal	0.9 (n.c.)	1.0 (n.c.)	Normal
L	Jul-2017	M	24.7	pass	pass	Not complete (pupils too small)	NA	NA	NA	NA	NA	NA	NA

AABR: automated auditory brainstem response, SVS: Spot Vision Screener, n.c.: non-corrigible, NA in a shaded cell: not examined.

**Table 3:** Development of children with congenital Zika virus infection.

				Examinations at 23–25 Months of Age											Examinations at 51–62 Months of Age								
				Milestones		ASQ-3						ASQ:SE-2		*		ASQ-3						ASQ:SE-2	
ID	Month of Birth	Sex	Age (m)	Sitting without Support	Walking Alone	Communication	Gross Motor	Fine Motor	Problem Solving	Personal-Social	Overall	Social-Emotional	Overall		Age (m)	Communication	Gross Motor	Fine Motor	Problem Solving	Personal-Social	Overall	Social-Emotional	Overall
A	Jul 2017	F	24.7	**10**	13	P	**M**	P	P	P	Concern	P	concern	1	62.8	P	P	P	P	P	no	P	no
B	Aug 2017	M	23.9	6.5	12	P	P	P	P	**M**	Concern	P	concern	0	61.9	P	P	P	P	P	concern	P	concern
C	Aug 2017	M	25.0	6.5	12	P	P	P	P	P	No	P	no	0	61.8	P	P	**M**	P	P	concern	**M**	concern
D	Sep 2017	F	24.6	7	10	P	P	P	P	P	Concern	P	concern	1	NA	NA	NA	NA	NA	NA	NA	NA	NA
E	Sep 2017	M	25.2	8	12	P	P	P	P	P	No	**R**	no	1	61.3	P	**R**	P	P	P	no	P	no
F	Oct 2017	F	24.2	8	13	P	P	P	P	P	Concern	P	no	0	NA	NA	NA	NA	NA	NA	NA	NA	NA
G	Nov 2017	M	25.2	6	12	P	P	P	P	**M**	Concern	P	no	1	59.5	P	**R**	**R**	**M**	P	concern	P	concern
H	Nov 2017	F	25.0	6	12	P	P	P	P	P	Concern	P	concern	1	NA	NA	NA	NA	NA	NA	NA	NA	NA
I	Sep 2017	F	25.4	**8.5**	11	P	P	**M**	P	P	Concern	P	no	0	NA	NA	NA	NA	NA	NA	NA	NA	NA
J	Jul 2018	M	24.8	7	12	**R**	P	P	**R**	**M**	Concern	**R**	no	0	51	P	**M**	P	P	P	concern	**M**	concern
L	Jul 2017	M	24.7	**15**	**Not yet**	**R**	**R**	**R**	**R**	**R**	Concern	**R**	concern	15	NA	NA	NA	NA	NA	NA	NA	NA	NA

ASQ-3: Ages and Stages Questionnaire^®^, third edition, ASQ:SE-2: Ages and Stages Questionnaires^®^: Social-Emotional, Second Edition, * Modified Checklist for Autism in Toddlers, Revised, with Follow-Up™, P: pass (<1 standard deviations below the mean in ASQ-3 or below the 65th percentile point in ASQ:SE-2), R: referral zone (≥2 standard deviations below the mean in ASQ-3 or above the cutoff in ASQ-SE:2), M: monitoring zone (≥1 and <2 standard deviations below the mean in ASQ-3 or from the 65th percentile to the cutoff), NA in a shaded cell: not examined.

## Data Availability

The database of the study can be available upon contacting the corresponding author of this paper.
